# An Optimized Encryption Storage Scheme for Blockchain Data Based on Cold and Hot Blocks and Threshold Secret Sharing

**DOI:** 10.3390/e26080690

**Published:** 2024-08-15

**Authors:** Dong Yang, Wei-Tek Tsai

**Affiliations:** 1 School of Computer Science and Engineering, Beihang University, Beijing 100191, China; 2 College of Computer and Data Science, Fuzhou University, Fuzhou 350116, China; tsai@tiandetech.com

**Keywords:** blockchain data, encrypted storage, threshold secret sharing, data security

## Abstract

In recent years, with the rapid development of blockchain technology, the issues of storage load and data security have attracted increasing attention. Due to the immutable nature of data on the blockchain, where data can only be added and not deleted, there is a significant increase in storage pressure on blockchain nodes. In order to alleviate this burden, this paper proposes a blockchain data storage strategy based on a hot and cold block mechanism. It employs a block heat evaluation algorithm to assess the historical and correlation-based heat indicators of blocks, enabling the identification of frequently accessed block data for storage within the blockchain nodes. Conversely, less frequently accessed or “cold” block data are offloaded to cloud storage systems. This approach effectively reduces the overall storage pressure on blockchain nodes. Furthermore, in applications such as healthcare and government services that utilize blockchain technology, it is essential to encrypt stored data to safeguard personal privacy and enforce access control measures. To address this need, we introduce a blockchain data encryption storage mechanism based on threshold secret sharing. Leveraging threshold secret sharing technology, the encryption key for blockchain data is fragmented into multiple segments and distributed across network nodes. These encrypted key segments are further secured through additional encryption using public keys before being stored. This method serves to significantly increase attackers’ costs associated with accessing blockchain data. Additionally, our proposed encryption scheme ensures that each block has an associated encryption key that is stored alongside its corresponding block data. This design effectively mitigates vulnerabilities such as weak password attacks. Experimental results demonstrate that our approach achieves efficient encrypted storage of data while concurrently reducing the storage pressure experienced by blockchain nodes.

## 1. Introduction

Data in blockchain cannot be deleted, so the data in blockchain can only increase and cannot be reduced, which brings huge storage pressure to blockchain and hinders its further development, making many blockchain applications difficult to put into practice [1]. Currently, there are some research solutions that attempt to solve the scalability problem of blockchain storage, which can be roughly divided into the following categories:

1. Collaborative Storage: This aims to form a whole by integrating multiple storage nodes with limited capacity so as to complete the functions of a full node in a blockchain system. Literature [2] provides a detailed introduction to a distributed storage load balancing scheme based on distributed hash table technology that effectively utilizes a subset of nodes in the Bitcoin network to construct a distributed hash table cluster. In this cluster, each node can play the role of a full node, and it verifies new transactions by referencing other nodes, thus significantly reducing the need to store the entire blockchain. Literature [3] then proposes an innovative concept called a consensus unit to address the problem of limited storage scalability in blockchain systems. By constructing consensus units, the scheme significantly reduces the storage space required by each node. Furthermore, literature [4] demonstrates the storage engine BFT-Store, which significantly enhances storage scalability by combining the Reed–Solomon coding technique with the Byzantine fault-tolerance (BFT) consensus protocol. In BFT-Store, nodes only need to store the encoded fragments of each data block rather than the complete ledger, thereby achieving a significant reduction in the storage consumption of each data block and achieving O(1) storage efficiency.
2. Lightweight node model: As a different existence form from full nodes, its distinctive feature is that it does not need to store a complete copy of the ledger. Compared with full nodes that need to synchronize all block data, lightweight nodes adopt a more efficient strategy. Among them, the simple payment verification (SPV) protocol, as the first lightweight node protocol proposed, provides a simplified method for resource-constrained nodes by only storing the block headers of all blocks to achieve transaction verification. However, as the blockchain block height continues to grow, the SPV lightweight node, although it only needs to store the block headers, will also face the challenge of saturation of its storage space. At the same time, the SPV lightweight node still needs to rely on the data support provided by full nodes during the transaction verification process. In view of this situation, the academic community has conducted in-depth exploration, and a new public chain protocol was proposed in literature [5] that is particularly suitable for devices with limited storage resources. The protocol innovatively requires resource-constrained nodes to only store fixed-length data that are unrelated to the block height, thus abandoning the traditional practice of storing the block headers of each block. This further improves the efficiency and feasibility of lightweight nodes.
3. IPFS-based off-chain storage mechanism: InterPlanetary File System (IPFS) is a peer-to-peer network primarily used for data storage and access. It abandons the traditional addressing method based on server location and instead assigns unique encrypted hashes to dissimilar storage files, achieving a content-based addressing mechanism [6]. Literature [7] introduces a distributed architecture based on blockchain, aiming to securely share event information between roadside units without relying on trusted third-party cloud servers. Literature [8] proposes an electronic health record secure storage system that combines IPFS and blockchain, aiming to ensure the confidentiality of patient records while maintaining their integrity. Additionally, the system implements a role-based access control mechanism to reduce the risk of malicious intrusion. Literature [9] designs an online review system based on the Ethereum blockchain and IPFS. The system stores reviews using IPFS hash addresses on connected computer nodes, enhancing trust between reviewers and service providers while improving the performance of the review system.
4. Cloud-based off-chain storage: Literature [10] provides a detailed description of a lightweight decentralized encryption cloud storage architecture named Yugula. The architecture utilizes convergent encryption technology to encrypt files and innovatively stores the hash value of the file on the blockchain, thus achieving the decoupling of label and encrypted file association. This innovative design allows all users to effectively query the encrypted data in the cloud environment. Literature [11] then proposes a solution to optimize the node storage burden, allowing each node to store the old blocks created earlier and with a lower query probability in the cloud, which significantly reduces the storage pressure on nodes and effectively avoids the potential risks of block overflow. Furthermore, literature [12] introduces the concept of transparent integrity auditing and builds a blockchain-based mechanism for the secure transparent elimination of duplicate data. The solution not only supports the deduplication of encrypted data but also grants users the ability to verify duplicate data on the cloud server, thus ensuring that the data content is not interfered with by non-owners.

The above literature has alleviated the storage pressure of blockchain to some extent, but they all consider the storage optimization from a single on-chain or off-chain angle. Therefore, this paper, based on the existing research, fully considers the storage optimization of both on-chain and off-chain and designs a blockchain data storage optimization scheme based on the combination of cold and hot blocks and cloud storage to solve the problem of the high storage pressure of blockchain.

In this era of information explosion and data proliferation, how to protect personal privacy while enabling free flow and efficient utilization of data has become a pressing challenge. In current public chain systems such as Bitcoin and Ethereum, the data on the chain are not encrypted and there is no corresponding access control mechanism. Users can query all transaction data, which is not conducive to the protection of personal privacy. For example, the de-anonymization research of blockchain addresses [13] mainly uses the Ethereum domain name server (ENS) to de-anonymize, and, by comparing the data sets of the ENS with those of other social software, the real information of users can be found. Similarly, such problems also exist in consortium chain systems, such as the widely used Hyperledger [14]. There is no encryption storage mechanism in the chain data of Hyperledger. In some blockchain applications [15,16,17], such as financial, medical, and government blockchain applications, the data on the blockchain may contain sensitive information, such as transaction amounts, personal identities, and confidential data. For example, in the medical blockchain, a patient’s medical records should only be accessible to the patient and the attending physician, not to everyone. In the government blockchain, there may be sensitive information that needs to be encrypted for storage. In the evidence blockchain, only authorized individuals can view the corresponding evidence information. In similar blockchain applications, data need to be encrypted for the privacy and protection of sensitive information. Therefore, encryption storage is one of the important means to protect the security of blockchain data.

Literature [18] describes an attribute-based searchable encryption (ABSE) and storage mechanism that stores secure index information on a blockchain and encrypted data in a distributed system (such as the InterPlanetary File System (IPFS)) to avoid single points of failure. Literature [19] describes a blockchain-based proxy re-encryption technology for the Internet of Medical Things (IoMT), detailing the process of initialization, encryption, re-encryption, generation of accumulators, and cryptographic verification mechanisms to ensure the security of medical data by encrypting the ciphertext twice. Literature [20] describes an end-to-end data protection model for personal health records (PHRs) based on blockchain, using fully homomorphic encryption (FHE) methods, distributed hash table (DHT) technology, and blockchain networks to separately store and distribute data and metadata to protect individual privacy. Literature [21] describes how to use the secure encryption technology of blockchain to protect data privacy and integrity in the cloud environment. An innovative method is proposed, combining AES encryption, cloud storage (such as AWS S3), and Ethereum smart contracts to enhance the security of data during transmission and storage.

The above literature has solved the problem of the encrypted storage of blockchain data to some extent, protecting individual privacy. However, some of the solutions have low encryption efficiency, and some do not address the issue of key preservation, making them vulnerable to weak password attacks and causing key leaks. In view of the problems with existing solutions, we propose a cryptographic storage scheme based on threshold secret sharing [22]. The scheme combines the characteristics of threshold technology and the Byzantine fault-tolerance idea, dividing the encrypted key of the blockchain data into multiple fragments and storing them in various nodes in the network. The public key of the asymmetric encryption algorithm is used to perform the secondary encryption and storage of the fragmented key, effectively increasing the cost for attackers to obtain blockchain data. Additionally, we employ an encryption scheme where each key corresponds to a block and store the keys and block data together, effectively preventing weak password attacks and related issues.

This paper aims to address two main issues. The first is the problem of excessive node storage load caused by the inability to reduce the length of the blockchain, and the second is the issue of privacy protection and data security in blockchain applications in the fields of medicine and government affairs. The main contributions of this paper are as follows:

1. We have proposed an optimized blockchain data storage architecture that combines cloud storage with cold and hot block strategies. A hot block refers to blocks that are frequently accessed and will be stored on the blockchain node while a cold block refers to blocks that are hardly accessed and are stored in cloud storage. By integrating on-chain and off-chain storage optimization methods, we have introduced a block heat assessment algorithm to evaluate the heat of block data, thereby reducing the storage burden on blockchain nodes.
2. We have proposed an encryption storage scheme based on threshold secret sharing that utilizes symmetric encryption algorithms to encrypt blockchain data. The scheme divides the encryption key into multiple fragments using threshold secret sharing technology and stores them in various nodes within the network. Furthermore, the fragmented key undergoes secondary encryption using the public key of an asymmetric encryption algorithm before being stored. This approach effectively increases the cost for attackers attempting to access blockchain data and mitigates issues such as weak password attacks.

The structure of the remaining sections of this paper is as follows: Section 2 provides a review of related work on data encryption storage in blockchain while Section 3 offers a detailed description of the storage optimization architecture based on hot and cold blocks. In Section 4, we present the specific algorithm flow for the block heat assessment algorithm, followed by a description of the encryption storage scheme based on threshold secret sharing in Section 5. Section 6 includes experimental analysis covering storage performance and system security. Finally, in Section 7, we provide a summary of this paper.

## 2. Related Work

### 2.1. Cloud Storage

Cloud storage [23] is a model of online storage over the Internet that allows users to store data on multiple virtual servers hosted by third-party providers rather than traditional dedicated servers. Cloud storage is based on the concept of cloud computing and involves aggregating a large number of storage devices over the network to form a unified storage resource pool. Users can access these storage resources through web service application programming interfaces (APIs) or web-based user interfaces. In reality, the data may be distributed across numerous server hosts, but users do not need to concern themselves with the specific location of data storage.

The architecture of a cloud storage system can be categorized into the storage layer, management layer, interface layer, and access layer. The storage layer serves as the fundamental infrastructure of the entire architecture and is composed of diverse types of storage devices. Abstracting these storage devices enables the implementation of various forms of data storage. The management layer functions as the central component of the architecture and facilitates collaboration among different storage devices through clustered or distributed file systems. It also oversees data maintenance through processes such as data compression, encryption, and backup. The interface layer constitutes the user-facing segment within the overall architecture and is safeguarded by mechanisms such as network access control, identity authentication, and access control to ensure secure data retrieval processes while offering users an array of functionalities. Lastly, the access layer represents the application interface within this architectural framework that provides corresponding services based on users’ specific requirements.

In this paper, we use cloud storage systems to store cold block data, which are block data that are not frequently accessed by users, and only store hot block data on the blockchain node to reduce the storage pressure on the blockchain node.

### 2.2. Threshold Secret Sharing

Threshold secret sharing is a cryptographic technique designed to ensure the security of information and distribute risks by dividing secret information into multiple parts and distributing them to different participants. The secret is divided in such a way that no single participant can recover the original secret, and only when a specified number of participants collaborate can the original message be reconstructed. A threshold scheme (t, n) involves dividing the secret into n parts distributed among different users such that knowledge of any t or more sub-secrets allows for the reconstruction of the original secret, while knowledge of fewer than t sub-secrets makes it impossible to determine the original secret.

Threshold secret sharing reduces the risk of secret leakage by dispersing the secret among multiple storage nodes, thereby mitigating the impact of attacks or damage to a single node. Even in cases where some participants encounter issues or are targeted, as long as the remaining number of participants meets or exceeds the threshold value t, it is still possible to fully recover the secret. The reconstruction of the initial secret requires at least t participants to collectively provide their respective sub-secrets, thus enhancing both the complexity and security of secret recovery. Currently, there are two main threshold secret sharing schemes:

1. The Shamir threshold secret sharing scheme [24]: By constructing a t − 1 degree polynomial and setting the secret as the constant term of the polynomial, the secret is divided into n parts and distributed to n participants.
2. Blakley’s threshold secret sharing scheme [25]: This utilizes points in a multi-dimensional space to establish a threshold scheme, where the shared secret is represented as a point in a t-dimensional space and each sub-secret corresponds to the equation of a (t − 1)-dimensional hyperplane containing this point.

This paper employs a threshold secret sharing scheme based on Shamir’s secret sharing algorithm for the distributed storage of encrypted keys.

### 2.3. Decentralized Identifiers

In the specification for distributed identifiers (DIDs) [26] released by the World Wide Web Consortium (W3C), a DID is defined as a new globally unique identifier. This identifier can be used not only for individuals but also for various entities such as vehicles, animals, and machines. The core components of DID technology include three elements: DID, DID documents, verifiable credentials, and verifiable presentation.

**Figure 1:**
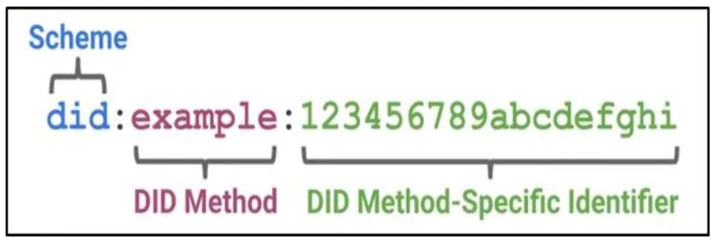
DID identification format.

A DID (decentralized identifier) is a specific format of string used to represent a digital identity for an entity, which can be a person, machine, or thing. The format of a DID is as follows:

As shown in Figure 1, the DID document contains all the information related to the DID identifier, is connected to the DID identifier through a uniform resource locator (URL), and is a universal data structure. It is usually responsible for data writing and modification by the DID controller. The file contains key information and verification methods related to DID verification and provides a set of mechanisms that enable the DID controller to prove its control over the corresponding DID identifier. The DID controller may be the DID identifier itself or a third-party institution. Different DID methods have different permissions management for DID Doc. The binding of user-centered identity and other identifiers issued by recognized institutions is called verifiable credentials (VCs). The DID document itself cannot be associated with the user’s real identity information, and VCs are needed to achieve this. The association process is the value of the entire system. VCs are similar to a digital certificate, which proves the user’s identity, and also provide a system similar to PKI. Verifiable presentation (VP) is the data used by the VC holder to demonstrate their identity to the verifier. In general, we can simply present the full text of the VC, but, in some cases, for privacy protection reasons, we do not want to present the full content of the VC and only want to disclose certain attributes or none at all, and only need to prove a certain assertion. In this paper, we use a DID for data privacy protection and access control.

## 3. Optimized Storage Architecture

The application of blockchain in different scenarios has enhanced the publicity, transparency, and immutability of data due to its high redundant storage mechanism. However, the data in blockchain cannot be deleted, so the data in blockchain can only increase and not decrease, which will bring huge storage pressure to the blockchain nodes. To alleviate the storage pressure of blockchain nodes, we propose a blockchain data encryption storage optimization scheme based on cold and hot blocks and threshold secret sharing. We introduce a cloud storage system to store infrequently used blocks, i.e., cold blocks. First, we describe the heat of each block comprehensively by extracting the block features in the blockchain and design a block heat evaluation algorithm to distinguish between hot blocks and cold blocks. The hot blocks are stored in the blockchain nodes, and the blockchain nodes only store the block headers and hash values of the cold blocks while the cold block data are stored in the cloud storage system. Then, to protect the security of blockchain data, we design a blockchain data encryption scheme based on threshold secret sharing. Due to the low encryption efficiency of asymmetric encryption algorithms, we use symmetric encryption algorithms to encrypt and store blockchain data. Due to the existence of Byzantine nodes in the blockchain system, there is a risk of symmetric encryption key leakage. We use threshold secret sharing to distribute the symmetric encryption key, and only after obtaining more than the threshold number of secret shares can the symmetric encryption key be restored, thus protecting the security of blockchain data.

The Pareto principle [27] was proposed by Italian economist Vilfredo Pareto. This principle indicates that there is an imbalanced relationship between causes and effects, inputs and outputs, and effort and rewards. Typically, 80% of outputs or rewards are generated by 20% of causes, inputs, or effort. It reminds us to focus on the key factors that can produce the greatest impact rather than spreading our energy evenly across all things. By prioritizing the important few, we can more effectively use resources, improve efficiency, and increase output. In blockchain, the Pareto principle also applies, first manifested in node distribution, where, in a blockchain network, only 20% of nodes may be active but these nodes handle 80% of transactions or data. This means that although there are many nodes in the network, the few nodes that really have a significant impact on network performance are only a small percentage of the total. In the Bitcoin network, there are only about 20% or fewer active nodes. The Pareto principle also applies to the use of blockchain data, where only about 20% of the transaction data are frequently queried in Bitcoin or Ethereum networks [28], which means that we do not have to store all the duplicate data in every blockchain node. We only store the frequently queried data in the blockchain nodes, and other data are stored in cloud storage.

**Figure 2:**
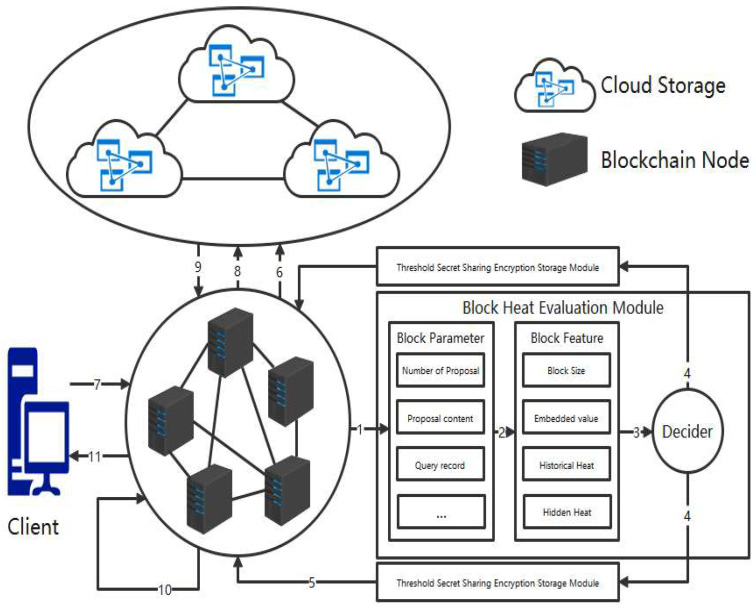
An optimized blockchain data storage architecture based on cold and hot blocks and threshold secret sharing.

As shown in Figure 2, our storage optimization architecture consists of cloud storage, a blockchain system, a client, a block heat evaluation module, and a threshold secret sharing encryption storage module. Cloud storage can be composed of blockchain-based cloud storage systems [29,30,31] or general cloud storage servers. Cloud storage is responsible for maintaining cold block data that have been offloaded from the blockchain system. The blockchain system consists of blockchain nodes, where each node stores the hash values of the commonly used hot block encrypted data and the cold block encrypted data. The client performs two operations: submitting data and querying data. The proposals submitted by the client undergo consensus, block building, and encryption within the blockchain system before being stored. Query requests submitted by the client are evaluated by blockchain nodes. If it is a hot block, the data are directly returned; if it is a cold block, data retrieval from cloud storage is required, followed by data integrity verification before returning the data. The block heat evaluation module determines whether a block belongs to the hot block and extracts block parameters to form block features, and then the decider makes a decision. The threshold secret sharing encryption storage module is responsible for encrypting and storing blockchain data. We use symmetric encryption algorithms for encrypting blockchain data. Due to the existence of Byzantine nodes in the blockchain system, there is a risk of symmetric encryption key leakage. We use threshold secret sharing schemes to distribute the symmetric encryption key in a distributed manner, and the symmetric encryption key can only be restored after obtaining more than the threshold secret shares. The optimized process for encrypting and storing blockchain data is as follows:

1. The parameters to be tracked within the n round of block generation in the blockchain system include the number of proposals contained in each block, the content of each proposal within a block, and the historical query records associated with each proposal within a block.
2. According to the block parameters, the block feature values are extracted, and the actual size, intrinsic value, historical heat, and hidden heat of each block are calculated based on the block parameters statistically obtained in Step 1. The definitions and calculation methods of the four feature values are described in Section 2.
3. Determine the heat level of the block, set a heat threshold value, select an appropriate set of feature weight coefficients based on the needs of the actual application scenario, calculate the comprehensive heat level of the block based on the evaluation algorithm, and make a heat judgment. Blocks with heat levels exceeding the heat threshold are considered hot blocks, while those below the threshold are considered cold blocks.
4. After the block heat judgement is completed, the judgement results are fed back to the blockchain system, and the blocks with combined heat exceeding the heat threshold are considered hot blocks; otherwise, they are cold blocks.
5. Data are encrypted using a symmetric encryption algorithm, and the symmetric encryption key is distributed for storage using a threshold secret sharing scheme.
6. Blocks that are judged by the decider to be cold are offloaded to the cloud storage system and only the block header and the hash value of the encrypted data are stored in the blockchain node. The storage location of the cold block is determined by the consistent hash algorithm based on the hash value of the block so that the client can easily query the cold block data.
7. The client sends a query request to retrieve data within a block, but since there may be Byzantine nodes in the blockchain system, the query request needs to be broadcast to all the blockchain replica nodes. Let us assume that there are n nodes in the blockchain system, and f of them are Byzantine nodes, where f < n/3. The client can consider that it has received the correct data once it receives f + 1 identical feedback results.
8. Each node in the blockchain system receives a query request from the client and performs a judgment. If the query content is within the hot block, it jumps to step 11 and returns the query result. If the query content is within the cold block, it calculates the location where the cold block is stored using the consistency hash algorithm to obtain the encrypted data of the cold block.
9. When a cloud storage receives a request from a blockchain replica node to retrieve cold block encrypted data, it returns the cold block encrypted data.
10. In the blockchain system, the integrity of cold block encrypted data is verified internally, and only after the data integrity verification is completed can the data be returned to the client.
11. In a blockchain system, each replica node uses a symmetric encryption key to decrypt the data and then feeds the decrypted data back to the client. The client can consider that it has received the correct data once it receives f + 1 identical feedback results.

## 4. Block Heat Evaluation Algorithm

We utilize a block heat evaluation algorithm to evaluate the heat of a block from four dimensions: block size, block value, block historical heat, and block correlation heat. Below is an introduction to the block heat evaluation algorithm.

We define the following features:

– Block size (BS);
– Block value (BV);
– Block historical heat (BHH);
– Block correlation heat (BCH).

These features collectively determine the heat of a block. The specific characteristics of the block heat assessment algorithm may vary for different applications based on blockchain at higher levels. In this article, we can redefine or modify other block features within the storage optimization architecture defined here. We will provide detailed definitions for each feature using an example of a blockchain system generating the n-th block after the n-th proposal.

### 4.1. Block Size

We tend to offload larger and less frequently queried blocks to cloud storage to reduce the storage pressure on the blockchain replica nodes, but it will bring greater query delay when querying the block. Since the focus of this solution is to alleviate the storage pressure of the blockchain replica nodes, larger blocks are more likely to be stored in cloud storage. We define the block size BS as

(1) $$BS={\left(BHS+BBS\right)}^{-1},$$

In this context, BHS stands for the size of the block header and BBS stands for the size of the block body. In a blockchain system, the size of the block header is often fixed, and the main difference in block size lies in the block body. The size of the block body equals the sum of the sizes of all proposals or transactions contained within the block. For example, if a particular block contains j proposals, the size of the block can be further expressed as

(2) $$BS={\left(BHS+\sum_{k=1}^{j}P_{k}\right)}^{-1},$$

where P_k_ represents the size of the kth proposal within the block body of that district.

### 4.2. Block Value

Taking financial applications using blockchain as an example, the value of a block can be represented as the sum of transaction amounts within the block and the product of the transaction breadth. A transaction primarily consists of three parts: the initiator, recipient, and amount. Let us assume that there are j transactions within a block; we can represent this set of transactions as T:

(3) $$T=(T_{1},T_{2},\ldots T_{j}),$$

where T_i_ represents the i-th transaction within the block, the transaction T_i_ can be represented as:

(4) $$T_{i}=({TS}_{i},{TR}_{i},{TV}_{i}),$$

In this context, TS_i_ represents the initiator of the i-th transaction within the block, TR_i_ represents the recipient of the i-th transaction within the block, and TV_i_ represents the transaction amount of the i-th transaction within the block.

The transaction breadth within a block is defined as the proportion of initiators and recipients involved in transactions relative to the total number of registered users in the blockchain system. This can be expressed as the ratio of the union set of initiators and recipients to the total number of registered users. Let the number of registered users in the blockchain system be RU (registered users); then, the block value BV can be expressed as

(5) $$BV=\frac{1}{j}\sum_{k=1}^{j}{TV}_{k}\cdot \frac{TS\cup TR}{RU},$$

TS represents the set of all transaction initiators within a block while TR represents the set of all transaction receivers within a block. $\frac{1}{j}\sum_{k=1}^{j}{TV}_{i}$ denotes the average value of transactions within a block. It is necessary to calculate the average value of the sum of all transactions within a block because a block may contain numerous low-value transactions, leading to an overall high block value. However, such blocks are relatively less likely to be targeted by malicious nodes (as these nodes typically tend to honestly handle low-value transactions and attack high-value ones). Therefore, taking the average value serves as an assessment dimension for the heat level of a block.

In non-financial blockchain applications, the data value of proposals within a block can be used instead of transaction amounts in financial blockchain applications to represent the final value of a block.

### 4.3. Block Historical Heat

The historical records of block queries from the client side also impact the heat of a block. By analyzing the historical query records of a block, its historical heat can be evaluated based on two aspects: query frequency and query time. When a block is frequently queried by clients, it should be stored in the copy nodes of the blockchain to facilitate future queries. Furthermore, if a block has a small total query volume but has been queried multiple times recently, it can still be considered as a hot block. If we denote the total number of queries on a block as n, then the historical heat of the block can be represented as follows:

(6) $$BHH=\sum_{q=1}^{n}{\left(T_{q}\right)}^{-1},$$

In this context, T_q_ denotes the time difference between the current moment and the qth query for this block.

### 4.4. Block Correlation Heat

In some cases, the historical heat of a block may not be high, but if the user who submitted the block is involved in data stored in other blocks that have been frequently queried recently, it means that the block may also be queried in the future, which indicates that the block’s associated block has a higher block correlation heat if it is frequently queried.

For example, in the case of a blockchain application in the financial sector, let TS_m_ be the set of transaction initiators in block m, TR_m_ be the set of transaction recipients in block m, TS_j_ be the set of transaction initiators in block j, and TR_j_ be the set of transaction recipients in block j. Then, the block correlation coefficient between block m and block j can be defined as

(7) $$\theta_{mj}=\frac{{TS}_{m}\cap {TS}_{j}+{TR}_{m}\cap {TR}_{j}}{{TS}_{j}\cup {TR}_{j}},$$

If the number of blocks associated with block m is n, then the correlation heat BCH of block m can be represented as

(8) $${BCH}_{m}=\sum_{j=1}^{n}{BHH}_{j}\cdot \theta_{mj},$$

Therefore, the feature set FS of n blocks can be represented as a 4xn matrix:

(9) $$FS=\left[\begin{array}{c} \begin{array}{ccc} {BS}_{1} & {BV}_{1}{BHH}_{1} & {BCH}_{1}\\ {BS}_{2} & {BV}_{2}{BHH}_{2} & {BCH}_{2}\\ \vdots & \vdots \;\;\;\;\;\;\;\;\;\vdots & \vdots \end{array}\\ \begin{array}{ccc} {BS}_{n} & {BV}_{n}{BHH}_{n} & {BCH}_{n} \end{array} \end{array}\right],$$

The comprehensive heat index BH of a block is defined as

(10) $$BH=\alpha_{1}\cdot BS+\alpha_{2}\cdot BV+\alpha_{3}\cdot BHH+\alpha_{4}\cdot BCH,$$

In this model, BW = ($\alpha_{1},\alpha_{2}$,$\alpha_{3}$,$\alpha_{4}$) represents the weight coefficients of each block feature, which must satisfy the constraint $\alpha_{1}+\alpha_{2}+\alpha_{3}+\alpha_{4}=1$, and $\alpha_{1},\alpha_{2}$,$\alpha_{3}$,$\alpha_{4}\in [0,1]$. The decision matrix of the block BD is

(11) $$BD=FS\cdot {BW}^{T},$$

Blocks with a combined heat BH not less than the decision threshold DT are considered as hot blocks and stored within the replica nodes of the blockchain. It is important to note that the decision threshold DT should be relevant to the actual application. The algorithm for evaluating block heat is presented in pseudocode form below, as detailed in Algorithm 1.
**Algorithm 1:** Block Heat Evaluation AlgorithmInput: BLOCK = {B_1_, B_2_, …, B_n_}, BW = (a_1_, a_2_, a_3_, a_4_), RU, DTVOutput: BH = {BH_1_, BH_2_, …, BH_n_}1: initialization BS, BV, BHH, BCH, TS, TR2: for each B_i_ in BLOCK do3:      Record the block size BS_i_4:      Record the query time tq_i_5:      TQ ← {tq_1_, tq_2_, …, tq_n_}6:      T_now_ ← time now7:      for each T_j_ in B_i_ do8:           Record the trade value TV_j_9:           Record the trade sender TS_j_10:         Record the trade receiver TR_j_11:    end for12:    TS_i_ ← {TS_1_, TS_2_, …, TS_j_}13:    TR_i_ ← {TR_1_, TR_2_, …, TR_j_}14:    ${\mathrm{BV}}_{i}\;\leftarrow \;\frac{1}{j}\sum_{k=1}^{j}{TV}_{k}\cdot \frac{{TS}_{i}\cup {TR}_{i}}{RU}$
15:    ${\mathrm{BHH}}_{i}\;\leftarrow \;\sum_{q=1}^{n}{\left(T_{now}-{TQ}_{q}\right)}^{-1}$
16: end for17: for each B_m_ in BLOCK do18:      for each B_j_ in BLOCK do19:           if m! = j then20:               $\theta_{\mathrm{mj}}\;\leftarrow \;\frac{{TS}_{m}\cap {TS}_{j}+{TR}_{m}\cap {TR}_{j}}{{TS}_{j}\cup {TR}_{j}}$
21:           end if22:      end for23:      ${\mathrm{BCH}}_{m}\;\leftarrow \;\sum_{j=1}^{n}{BHH}_{j}\cdot \theta_{mj}$
24: end for25: F S ← [BS, BV, BHH, BCH]26: BH ← FS · BW^T^27: for i = 1 to n do28:      if BHH_i_ >= DT V then29:          BLOCK_i_ is a hot block30:      else31:          BLOCK_i_ is a cold block32:      end if33: end for

## 5. An Encryption Storage Scheme Based on Threshold Secret Sharing

Encrypted storage can ensure that data on the blockchain are not accessed or tampered with by unauthorized third parties during storage and transmission. By encrypting the data, even if they are stolen or leaked, attackers will find it difficult to decrypt and obtain the original information. This helps to protect users’ privacy and data security while also helping to maintain the integrity and credibility of the blockchain system. In addition to privacy protection, encrypted storage can also prevent data abuse. In some cases, data on the blockchain may be used for illegal or immoral purposes, such as identity theft and fraud [32]. By encrypting storage, access and use of these data can be limited, thereby reducing the risk of data abuse.

Current data encryption algorithms include symmetric encryption algorithms and asymmetric encryption algorithms. Symmetric encryption algorithms include AES and DES, which have high encryption efficiency, but the ciphertext can be easily cracked, and the encryption and decryption key is the same, so it is easy to leak the key when encrypting and decrypting data on non-trusted nodes. Asymmetric encryption algorithms can solve this problem. Common asymmetric encryption algorithms include the RSA algorithm, DSA algorithm, and elliptic curve encryption algorithm. Asymmetric encryption requires two keys: a public key and private key. The public key and private key are a pair. If the data are encrypted with the public key, they can only be decrypted with the corresponding private key. If the data are encrypted with the private key, they can only be decrypted with the corresponding public key. Because encryption and decryption use different keys, it is called asymmetric encryption. Asymmetric encryption has good confidentiality and eliminates the need for end users to exchange keys. However, the encryption and decryption speed are much slower than symmetric encryption, and, in some extreme cases, they can be up to 1000 times slower than symmetric encryption [33].

The current mainstream solution for encrypting blockchain data involves using symmetric encryption algorithms to encrypt block data and employing asymmetric encryption schemes to store and transmit the encryption keys. However, the storage and management of encryption keys predominantly rely on centralized solutions. This can lead to the collapse or unavailability of the blockchain encrypted storage system in cases where the management node is attacked or experiences downtime, and it also makes it susceptible to weak password attacks [34]. For instance, in contemporary blockchain wallets, private keys are commonly stored using an encrypted file format known as Keystore to prevent asset loss. Keystore represents the most widely used private key storage file, with a data structure based on JSON format encrypted with a user-defined password. Blockchain wallet users can recover and utilize their private key data through custom passwords via Keystore files. While storing private keys in Keystore files facilitates convenient and swift transaction signing, its protection solely relies on user-defined password encryption, making it highly vulnerable to weak password attacks. Once compromised, access to private key information, including account addresses, poses a serious threat to asset security for users.

### 5.1. Scheme Design

Due to the fact that data are not encrypted and stored in traditional blockchain systems, they are almost publicly available, allowing almost anyone to access users’ on-chain data, which is not conducive to protecting users’ privacy. Even if data are encrypted and stored, since blockchain system nodes save almost all blockchain data, if a single point of attack occurs and the encryption key is stolen, it will also cause data leakage. This chapter aims to solve the problems of encrypted data storage in blockchain systems by proposing a distributed blockchain data encryption storage scheme based on threshold secret sharing technology. The scheme combines the characteristics of threshold technology and the Byzantine fault-tolerance idea and divides the encryption key of the blockchain data into multiple fragments and stores them in various nodes in the network. Users in the blockchain network can request to retrieve more than the threshold value of the key fragments to recover the key and decrypt the data. The scheme can effectively increase the attacker’s attack cost of obtaining blockchain data.

We adopt a one-to-one mapping of blocks to keys for encrypting blockchain data. This is because using the same key for all data encryption makes the key vulnerable to being cracked. For each block, we employ a different key for encryption. Considering the efficiency of data encryption, asymmetric key encryption is less efficient. Therefore, we use symmetric encryption algorithms for encrypting block data, with AES being a commonly used algorithm in this context. The symmetric encryption keys are encrypted using asymmetric encryption methods. Storing keys at a single point can lead to issues such as key leakage or system failure; hence, we need to distribute the encrypted keys using a threshold secret sharing scheme based on Shamir’s secret sharing algorithm [35].

**Figure 3:**
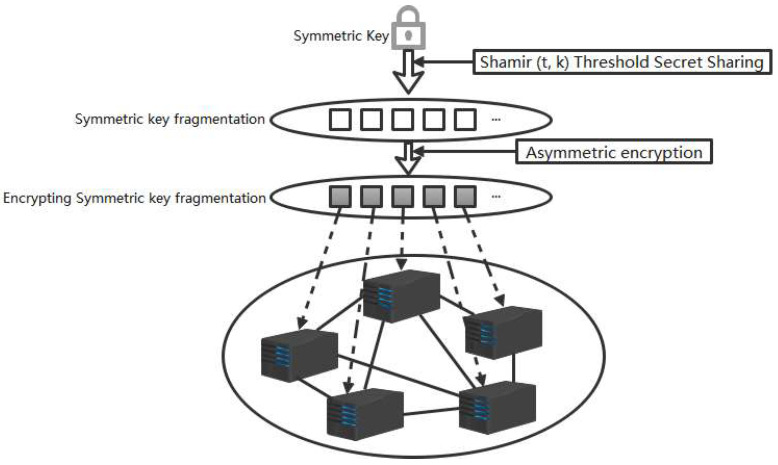
Diagram of symmetric key split strategy execution.

The diagram in Figure 3 illustrates the execution of the symmetric key split strategy.

We employ symmetric key encryption for the storage of block data to ensure the security of blockchain data. To safeguard the security of the symmetric keys, we utilize a scheme where each block is encrypted using a unique key. Furthermore, in order to protect the confidentiality of these keys, we apply a threshold secret sharing scheme based on Shamir’s secret sharing algorithm to fragment the symmetric keys into multiple shares. Subsequently, each secret share is encrypted using an asymmetric encryption algorithm’s public key and distributed for decentralized storage at specific locations within each block’s header. The encrypted symmetric key can only be decrypted by utilizing the private key of the block creator for key recovery operations. The symmetric key is generated randomly by the client or block creator, and a new symmetric encryption key is generated for every block. The secondary encryption of the key is encrypted using the block creator’s public key, and others that need to obtain the data must obtain authorization from the block creator. For detailed information on our threshold-secret-sharing-based blockchain data encryption algorithm, please refer to Algorithm 2 presented in pseudocode format below.
**Algorithm 2:** Encryption Algorithm based on Secret SharingInput: s, t, n, E, k, G, HOutput: C1:      initialization a_i_ = {a_0_, a_1_, …, a_t−1_}, u_j_ = {u_1_, u_2_, …, u_n_}2:      u_j_ = random(), j = 1, 2, …, n3:      Construction t−1 polynomial f(x) = s + $\sum_{i=1}^{t-1}a_{i}x^{i}$
4:      $p_{j}=f(u_{j})=s+\sum_{i=1}^{t-1}a_{i}u_{j}^{i}$, j = 1, 2, …, n5:      P_j_ ← (u_j_, p_j_)6:      M_j_ = Encode(P_j_)7:      Q = k · G8:      r = random(0, H)9:      C1_j_ = M_j_ + r · Q10:    C2_j_ = r · G11:    C = {(C1_1_, C2_1_), (C1_2_, C2_2_), …, (C1_n_, C2_n_)}

In this algorithm, s is the symmetric key used to encrypt the block data, t is the threshold value of the secret sharing scheme, n is the number of nodes in the blockchain system, E represents the elliptic curve encryption algorithm’s elliptic curve, k is the private key of the elliptic curve encryption algorithm, G is a point chosen on the elliptic curve as the generator, and H is the order of G. The output C is n encrypted symmetric key fragments that will be distributed to the nodes in the blockchain system. The specific steps of the algorithm are as follows.

Construct a threshold secret sharing scheme for [t,n], where n is the number of nodes in the blockchain system. For a given threshold t, users can use ciphertext s to construct a (t − 1)-degree polynomial f(x). Then, they select n pairs of (x, f(x)) and distribute them. By combining t points, one can solve for the coefficients of the polynomial, with the constant term representing the secret.

First, a symmetric key is generated, and the symmetric key s is computed using threshold technology. In this study, the threshold technology employs the Shamir secret sharing algorithm, where secret sharers randomly construct a t − 1 degree polynomial:

(12) $$f\left(x\right)=s+\sum_{i=1}^{t-1}a_{i}x^{i},$$

Substituting any n numbers into the above equation yields

(13) $$p_{j}=f\left(u_{j}\right)=s+\sum_{i=1}^{t-1}a_{i}{u_{j}}^{i},j=1,2,\ldots n,$$

Let $P_{j}=(u_{j},p_{j})$ be n fragments of the symmetric key s. The encryption and decryption of key fragments are performed using elliptic curve asymmetric encryption algorithms, constructing an elliptic curve Ep(a, b), where p is a prime number and x,y ∈ [0, p − 1]:

(14) $$y^{2}=x^{3}+ax+b\left(modp\right),$$

Among them, the curve is required to be non-singular (differentiable everywhere), and condition $4a^{3}+27b^{2}\neq 0$ must be satisfied. A point G on the curve is chosen as the generator, and the order of G is denoted as H. It is required that H must be a prime number. Based on the private key k (k < H), the public key Q = kG is generated. The symmetric key is divided into segments P_j_, which are encoded as M_j_. M_j_ is a point on the elliptic curve, and a random number r (r < H) is selected. The two ciphertexts C1_j_ and C2_j_ are calculated as follows:

(15) $${C1}_{j}=M_{j}+rQ\;{C2}_{j}=rG\;C=\{({C1}_{1},{C2}_{1}),({C1}_{2},{C2}_{2}),\ldots ({C1}_{n},{C2}_{n})\},$$

The final encrypted key fragment is $C_{j}=\left({C1}_{j},{C2}_{j}\right)$, and Cj is distributed to various nodes within the blockchain system for storage.

The following is a pseudocode representation of the decryption algorithm for blockchain data based on threshold secret sharing. Please refer to Algorithm 3 for details.
**Algorithm 3:** Encryption Algorithm based on Secret SharingInput: C = {(C1_1_, C2_1_), (C1_2_, C2_2_), …, (C1_n_, C2_n_)}, kOutput: s1:      After collecting t secret key fragments2:      M_j_ = C1_j_ − k · C2_j_, j = 1, 2, …, t3:      P_j_ = Decode(M_j_)4:      (u_j_, p_j_) ← P_j_5:      p_j_ = a_0_$+a_{1}\;\cdot \;u_{j}+\ldots +a_{t}{-}_{1}\;\cdot \;u_{j}^{t-1}$
6:      $\left[\begin{array}{ccc} 1 & \cdots & u_{1}^{t-1}\\ \vdots & \ddots & \vdots \\ 1 & \cdots & u_{t}^{t-1} \end{array}\right]\left[\begin{array}{c} a_{0}\\ \cdots \\ a_{t-1} \end{array}\right]=\left[\begin{array}{c} p_{1}\\ \cdots \\ p_{t} \end{array}\right]$
7:      Solving matrix A = {a_0_, a_1_, … a_t−1_}8:      s = a0

The input C represents the encrypted key fragments stored by each node, while k denotes the private key of the elliptic curve asymmetric encryption and decryption algorithm. The output s corresponds to the symmetric encryption key for the encrypted block data. The specific steps of the algorithm are as follows:

After collecting t encrypted key fragments, key recovery can be performed by decrypting each key fragment using the private key of the block creator:

(16) $$M_{j}={C1}_{j}-k\cdot {C2}_{j},j=1,2,\ldots t,$$

where k is the private key of the block creator. Decode $M_{j}$ to obtain the secret key fragment $P_{j}$, use matrix multiplication to recover the secret key information for the secret key fragment $\left(P_{1},P_{2},\ldots P_{t}\right)$ that has reached the threshold number, and construct a polynomial according to $P_{j}=(u_{j},p_{j})$ for t participants:

(17) $$\left\{\begin{array}{c} p_{1}=a_{0}+a_{1}\cdot u_{1}+\ldots +a_{t-1}\cdot {u_{1}}^{t-1}\\ \begin{array}{c} p_{2}=a_{0}+a_{1}\cdot u_{2}+\ldots +a_{t-1}\cdot {u_{2}}^{t-1}\\ \ldots \end{array}\\ p_{t}=a_{0}+a_{1}\cdot u_{t}+\ldots +a_{t-1}\cdot {u_{t}}^{t-1} \end{array}\right.,$$

Obtain the following matrix:

(18) $$\left[\begin{array}{ccc} 1 & \cdots & {u_{1}}^{t-1}\\ \vdots & \ddots & \vdots \\ 1 & \cdots & {u_{t}}^{t-1} \end{array}\right]\left[\begin{array}{c} \alpha_{0}\\ \ldots \\ \alpha_{t-1} \end{array}\right]=\left[\begin{array}{c} p_{1}\\ \ldots \\ p_{t} \end{array}\right],$$

**Figure 4:**
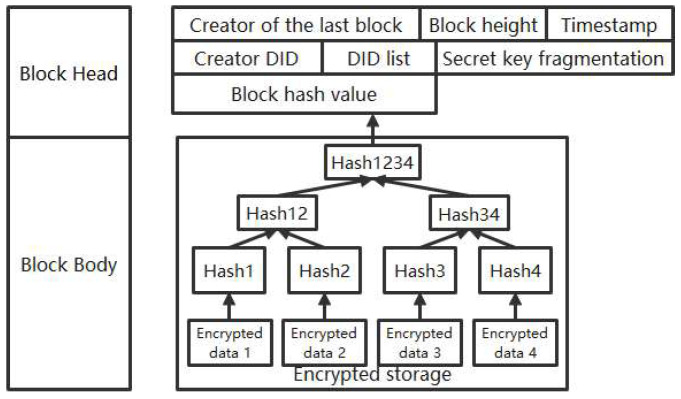
The data structure for encrypted storage of block data.

Using the matrix, the coefficients $\alpha_{0},\alpha_{1},\ldots \alpha_{t-1}$ are obtained, and the symmetric key $s=\alpha_{0}$ is obtained by substituting x = 0 into the polynomial f(x). The data structure for encrypted storage of block data is illustrated in Figure 4.

Block data consist of a block header and a block body. The block header includes the hash value of the previous block, the block height, timestamp, creator’s DID, DID list, and secret key fragments. The hash value of the previous block, block height, timestamp, and the hash value itself are commonly used to construct a block. The creator’s DID contains the identifier of either the creator or owner of the data in the block, signifying ownership. The DID list contains identifiers for those authorized to access the block along with their public keys; this list can be modified as needed. Secret key fragments store encrypted symmetric key segments used for encrypting data within the block body. A threshold secret sharing scheme is employed to divide symmetric keys into segments that are then encrypted using the public key of the creator before being distributed for storage in a decentralized manner.

The block body is composed of encrypted data and utilizes the Merkle tree structure for storing block body data. It employs symmetric key encryption for transactions within the block body. The Merkle tree is a tree-like data structure built on hash functions, and it exclusively operates on leaf nodes. The leaf nodes of the Merkle tree do not possess sibling nodes. Upon removing all leaf nodes from the Merkle tree, a complete binary tree can be obtained. Apart from the leaf nodes, all node values are either the hash value of their child nodes or the hash value of their child nodes’ sum.

### 5.2. The Process of Encrypting and Storing Blockchain Data

**Figure 5:**
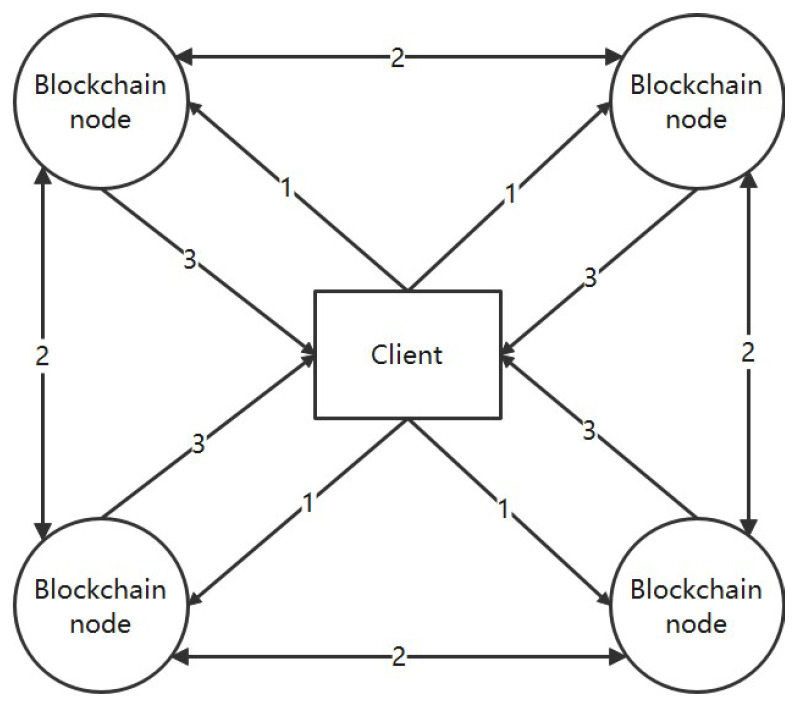
The process of encrypting and storing blockchain data.

We use DID for data privacy protection and access control in this study. We ignore the process of issuing DID and focus on the authentication process of obtaining encrypted data based on DID. The control over the submission of data by clients to the chain belongs to the data submitter. Other users need to obtain authorization from the data submitter to access the chain data. Figure 5 shows the flow of encrypting and storing blockchain data with DID authentication:

1. The client submits a proposal, and the information that the client needs to submit includes transaction information, the DID identifier of the user, a list of DID identifiers that allow for access to the transaction data, and a list of public keys, where the transaction information is the data that the client needs to store on the blockchain for the nodes to reach consensus and store, the DID identifier of the user will be assigned to the creator DID field in the block header data, which represents the ownership of the data, the list of DID identifiers that allow access to the transaction data will be assigned to the DID list field in the block header data, which represents the DID identity authentication information of the other users who are allowed to access the data, the list of public keys includes the public key of the user submitting the transaction and the public key of the other users who are allowed to access the transaction data, and the user submitting the transaction can update the DID identifier list and the list of public keys data. Because there may be Byzantine nodes in the blockchain system, in order to ensure the effectiveness of the on-chain operation, the client needs to broadcast this information to all blockchain nodes.
2. The blockchain nodes achieve consensus on the information submitted by clients using consensus protocols such as PBFT and Hotstuff or QuickBFT [36]. Upon reaching consensus, the data are stored using the proposed encrypted storage scheme in this study, and the key is fragmented for distributed storage.
3. After the consensus and storage of blockchain nodes are completed, the results are fed back to the client. When the client receives more than f + 1 successful feedback, it is considered that the client’s proposal has been successfully recorded on the blockchain. The number of nodes in the blockchain is n = 3f + 1.

### 5.3. The Process of Obtaining Encrypted Data on a Blockchain

**Figure 6:**
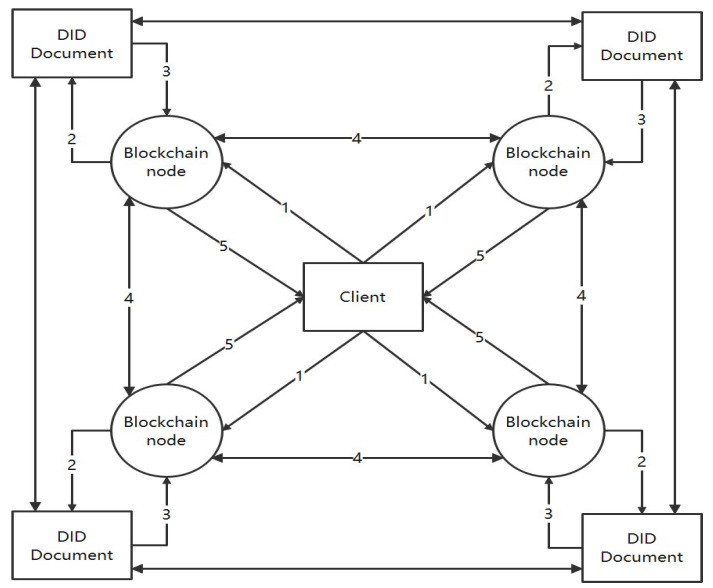
The process of data acquisition for blockchain encrypted data by users.

Figure 6 shows the data acquisition process for users to obtain encrypted data from blockchain:

1. Users submit data query requests, which require the following data to be submitted: data index information, user DID identifier, and the encrypted DID identifier using the user’s private key. The data index information is used to locate the block, including the query block height, block hash value, and the creator’s DID information. Due to the possible Byzantine nodes in the blockchain system, to ensure the effectiveness of data query operations, users need to broadcast data query requests to all blockchain nodes.
2. When a blockchain node receives a data query request from a user, it locates the specific block position based on the data indexing information, searches for the user’s DID identifier in the DID list in the block header, and skips to step 5 if it is not found. If it exists, it searches for the corresponding public key of the user in the DID list and decrypts the DID identifier encrypted by the user’s private key using the user’s public key. Then, it searches for the identifier in the DID document and verifies its authenticity. The DID document is stored in a distributed manner in a location that everyone can control, making it easy to find.
3. The DID document returns the search results, and if the user’s DID identifier does not exist, it jumps to step 5.
4. Blockchain nodes broadcast key fragments of the queried block, and nodes aggregate the key fragments and decrypt the block data based on the decryption scheme based on the threshold secret sharing described in this study.
5. If the DID identifier is not found in the DID list or the DID document does not contain the user’s DID identifier, the result will be a query failure. Otherwise, the block data to be queried will be returned.

## 6. Experimental Analysis

We conduct storage performance analysis for our research plan through simulation experiments, including experimental and analytical studies on storage space utilization and query hit rate. We also conduct theoretical analysis on system security, including efficiency analysis and data security analysis of encryption, analysis of resistance to weak password attacks, and analysis of resistance to collusion attacks.

In the simulation test, we used MATLAB (R2019a) to simulate the block-building and storage process of the blockchain system. The experimental environment was as follows: the processor was Intel Core i7-8550U, with a clock frequency of 1.8 GHz and 16 GB of memory, and the operating system was Microsoft Windows 10 64 bit. Since there was no real data set that met the experimental requirements (for example, we did not have the qualifications to access certain blockchain application platforms and could not obtain data; although we could obtain block data on public chain systems such as Bitcoin and Ethereum, we could not obtain information such as the query frequency of the blocks), we did not use a real data set in the experiment but used artificially generated data as the experimental data set.

### 6.1. Storage Performance Analysis

#### 6.1.1. Storage Load Test

We select the average storage load on the network as the performance indicator, and the comparison scheme is a full replication-type blockchain such as Bitcoin, Ethereum, and Hyperledger. The experimental results are the average of multiple runs. The average storage load on the network is the ratio of the total storage space occupied by all nodes to the number of nodes. We set the experimental parameters as shown in Table 1.

**Figure 7:**
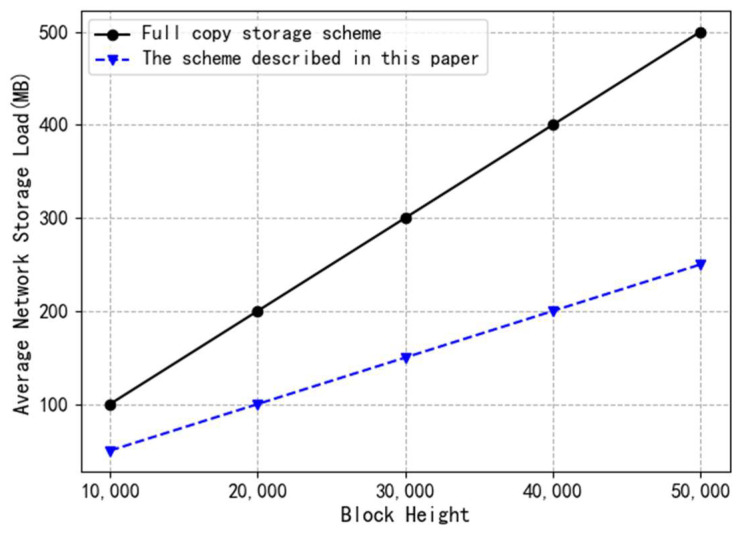
Storage load comparison experiment results.

The experimental results of the storage load experiment are presented in Figure 7. When maintaining a blockchain with a block height of 50,000 using the storage optimization scheme proposed in this paper, we compared the storage load for block heights of 10,000, 20,000, 30,000, 40,000, and 50,000. The average storage load of the entire network is significantly lower than that of the blockchain network using the full replication storage scheme. This is due to the fact that, in traditional full-replication-based blockchains, each node is required to store all blocks on the chain, leading to large storage space occupation by all nodes. In contrast, our proposed scheme only stores blocks with high activity while offloading other blocks to cloud storage for retention.

#### 6.1.2. Analysis of Block Query Efficiency

We select the hit rate as a performance indicator when querying a block. When querying a block, if the block is stored in the edge blockchain layer, it is called a hit, and if the block is offloaded to the cloud storage layer and a request needs to be made to the cloud storage layer, it is called a miss. We define the query hit rate as the ratio of hit queries to the total number of queries. Since hot blocks are usually more likely to be queried, i.e., the request probability of a block can be considered as the block’s popularity, we use the Zipf distribution to simulate the probability of requesting a block from the edge blockchain layer. The probability of requesting block bi (the i-th most popular block) is

(19) $$P_{i}=i^{\left(-\gamma \right)}/\sum_{j=1}^{h}j^{\left(-\gamma \right)},$$

where γ is the skew parameter of the Zipf distribution, with a larger value indicating a higher probability of requesting hot blocks. The total number of blocks is denoted by h. The comparison scheme is based on literature [37], and experimental results are obtained through multiple runs and averaging. Firstly, we compare the query hit rates for two storage schemes when maintaining the same blockchain but selecting different skew parameters for edge layer storage to validate the proposed storage optimization scheme’s advantage in query efficiency. Subsequently, we examine the impact of decision thresholds on improving query efficiency for the proposed storage optimization scheme. Experimental parameters are set as shown in Table 2.

**Figure 8:**
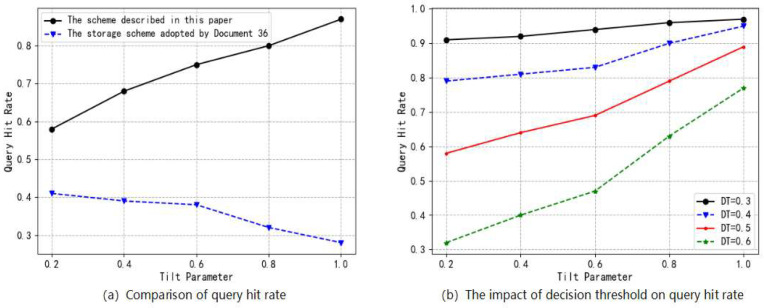
Experimental results of block query efficiency.

Figure 8a illustrates the comparison of query hit rates between the storage scheme proposed in this paper and the scheme outlined in [36]. The query hit rate of the storage optimization scheme proposed in this paper surpasses that of the storage scheme utilized in [36], with the disparity between them increasing as the skew parameter grows. This discrepancy arises from the fact that our proposed storage optimization scheme determines block hotness by extracting block features, enabling us to select blocks with higher hotness for local storage at the edge layer. In contrast, the storage scheme employed in [36] can only randomly select a portion of blocks for local storage after computing minimum replica numbers for each block within each round of consensus. As such, as requests for high-hotness blocks become more probable, local hit rates decrease. In Figure 8b, we present experimental results demonstrating how different decision thresholds impact query hit rates. When maintaining four distinct decision thresholds for a single blockchain, it becomes evident that lower decision thresholds result in higher edge layer query hit rates for our proposed storage optimization scheme. This relationship is due to the inverse proportionality between decision thresholds and the number of hot blocks identified per unit time; thus, when requesting a block, there is a greater likelihood that it is stored within an edge blockchain layer if using a lower threshold. Consequently, when facing significant storage pressure within an edge blockchain layer and exhibiting insensitivity to delays associated with block queries, setting a higher decision threshold allows for prioritization toward alleviating node-level pressures within these layers. Conversely, when encountering minimal storage loads within an edge blockchain network while remaining sensitive to delays related to block queries, setting a lower threshold takes precedence in reducing these delays.

#### 6.1.3. Analysis of Encryption Storage Efficiency

**Figure 9:**
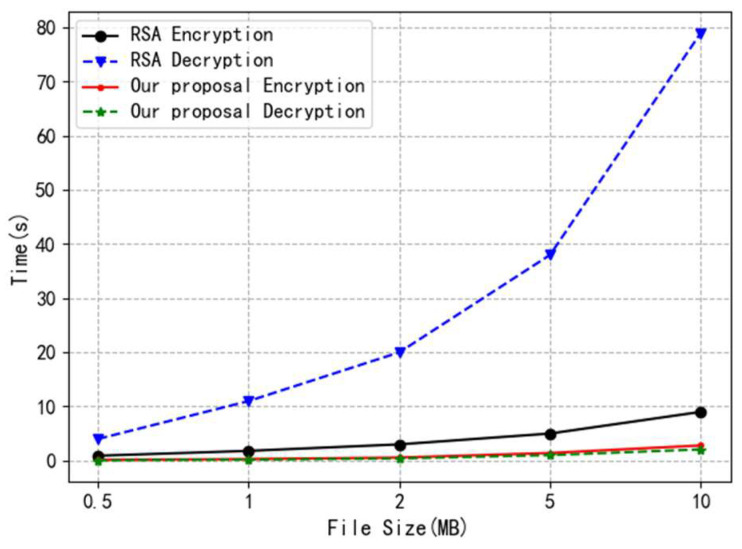
An experimental comparison of encryption efficiency.

We use a threshold-secret-sharing-based encryption storage scheme to split the encryption key of blockchain data into multiple fragments and store them on nodes in the network. We use an AES symmetric encryption key for the encryption and storage of blockchain data. Compared with asymmetric encryption algorithms such as the RSA algorithm, the main computational cost of the AES algorithm is the shift operation, while the main computational cost of asymmetric encryption algorithms is the exponentiation operation. The AES algorithm has faster encryption but slower decryption and has higher encryption and decryption efficiency. In the most extreme case, for example, when the size of the encrypted file is 1 G, the decryption time of the RSA algorithm, under the same configuration, will be 500 times that of the AES algorithm. We conducted an experimental comparison of the encryption efficiency of the encryption scheme described in this paper with the RSA algorithm, as shown in Figure 9. The experimental results show that the encryption efficiency of the encryption scheme described in this paper has a significant improvement over the RSA algorithm.

### 6.2. System Security Analysis

#### 6.2.1. Data Security Analysis

We employ a key-per-block encryption method to store block data and utilize threshold secret sharing to distribute the encryption keys in a distributed manner for key security protection. While the AES algorithm may be susceptible to brute-force attacks (using side-channel attacks), it is nearly impossible to decrypt the entire blockchain system’s data even if one block is compromised due to our approach of one key corresponding to one block. The security of AES encryption also depends on the management and implementation of the key. If the key management is not performed properly (e.g., the key is leaked or guessed), the encrypted data may be decrypted. In this study, the encryption key will not be stored in a single point but will be distributed using a distributed scheme based on threshold secret sharing for key fragmentation. This eliminates the possibility of key leakage. In terms of access control, we have adopted an access control mechanism based on DID distributed identity authentication to protect the data security of the blockchain.

#### 6.2.2. Analysis of Resistance to Weak Password Attacks

In the typical encryption storage of blockchain data, users’ passwords are stored locally or on the server end. Once a user’s local computer is compromised by an attacker or if the server is attacked, resulting in key leakage, the encrypted files become exposed to the attacker. For instance, when encrypted storage files utilize a user-defined secret passphrase, they are highly susceptible to weak passphrase attacks. This refers to passphrases that can be easily guessed by others through simple and common thought processes due to their composition of commonly used numbers, letters, and characters. Exploiting weak passphrases in combination with vulnerabilities in computer systems can lead to significantly amplified intrusion effects.

The system proposed in this paper implements a method where encryption keys are distributed randomly and in a decentralized manner across node networks based on secret sharing algorithms and then undergo encryption processing using elliptic curve asymmetric encryption algorithms. When attackers target individual nodes within the blockchain network, they are unable to access any data directly associated with the encryption keys.

#### 6.2.3. Analysis of Resistance to Collusion Attacks

In a distributed storage node network, collusion attack [38] refers to the presence of multiple malicious nodes that aggregate locally stored key fragments to decrypt keys. For instance, if an attacker compromises a distributed storage node network and acquires more than the threshold value (denoted as t) of key fragments x for a specific block, they can derive the encrypted key for that block through aggregation. However, in this study, the key fragments of the encrypted key are all encrypted using the user’s public key. As long as attackers cannot obtain the user’s private key, they will be unable to access any data stored by that user within the blockchain system.

## 7. Conclusions

This article presents an optimized blockchain data encryption storage scheme based on hot and cold blocks, as well as threshold secret sharing. This scheme aims to address the storage pressure problem of blockchain nodes and ensure secure data storage. It introduces a cloud storage system to store commonly encrypted block data in blockchain nodes while offloading cold block data to the cloud storage system. By employing a block heat evaluation algorithm that considers historical heat and correlation heat indicators, this approach effectively reduces the storage pressure on blockchain nodes. Additionally, this article proposes a cryptographic storage scheme based on threshold secret sharing that leverages both threshold technology characteristics and Byzantine fault-tolerance principles. This involves dividing the encryption key of blockchain data into multiple fragments stored across various network nodes, with each fragment further encrypted using a public key before being stored. This method significantly increases attackers’ costs when attempting to access blockchain data.

Furthermore, by utilizing an encryption scheme where one key corresponds to one block, storing both the key and block data together helps to mitigate vulnerabilities such as weak password attacks. Experimental results demonstrate that this approach performs well in alleviating the storage pressure of blockchain nodes, improving data query efficiency, and enhancing the encryption efficiency of blockchain data. Moreover, a comprehensive system security analysis is conducted to elucidate how this proposed blockchain data encryption storage scheme enhances overall data security.

## Figures and Tables

**Table 1:** Storage load experiment parameter setting.

Parameter Name	Parameter Value
Number of nodes	N = 16
Block height	BH = 50,000
Block size	BS = 10 KB
Weight coefficient	BW = (0.25, 0.25, 0.25, 0.25)
Judgment threshold	DT = 0.5

**Table 2:** Experimental parameter setting of block query efficiency.

Parameter Name	Parameter Value
Number of nodes	N = 16
Block height	BH = 5000
Block size	BS = 10 KB
Weight coefficient	BW = (0.25, 0.25, 0.25, 0.25)
Judgment threshold	DT = 0.5
Query times	QN = 50,000

## Data Availability

The authors are unable or have chosen not to specify which data have been used.

## References

[1] Yin B., Li J., She Y., Wei X. (2024). Reducing Storage Requirement in Blockchain via Node-Oriented Block Placement. IEEE Trans. Netw. Sci. Eng..

[2] Abe R., Suzuki S., Murai J. Mitigating Bitcoin Node Storage Size By DHT. Proceedings of the Asian Internet Engineering Conference, AINTEC 2018.

[3] Xu Z., Han S., Chen L. CUB, a Consensus Unit-Based Storage Scheme for Blockchain System. Proceedings of the 34th IEEE International Conference on Data Engineering, ICDE 2018.

[4] Qi X., Zhang Z., Jin C., Zhou A. (2021). A Reliable Storage Partition for Permissioned Blockchain. IEEE Trans. Knowl. Data Eng..

[5] Khor J.H., Sidorov M., Zulqarnain S.A.B. (2023). Scalable Lightweight Protocol for Interoperable Public Blockchain-Based Supply Chain Ownership Management. Sensors.

[6] Kang P., Yang W., Zheng J. (2022). Blockchain Private File Storage-Sharing Method Based on IPFS. Sensors.

[7] Dwivedi S.K., Amin R., Vollala S. (2021). Blockchain-Based Secured IPFS-Enable Event Storage Technique With Authentication Protocol in VANET. IEEE CAA J. Autom. Sin..

[8] Pilares I.C.A., Azam S., Akbulut S., Jonkman M., Shanmugam B. (2022). Addressing the Challenges of Electronic Health Records Using Blockchain and IPFS. Sensors.

[9] Onwubiko A., Singh R., Awan S., Pervez Z., Ramzan N. (2023). Enabling Trust and Security in Digital Twin Management: A Blockchain-Based Approach with Ethereum and IPFS. Sensors.

[10] Gochhayat S.P., Bandara E., Shetty S., Foytik P. Yugala: Blockchain Based Encrypted Cloud Storage for IoT Data. Proceedings of the IEEE International Conference on Blockchain, Blockchain 2019.

[11] Xu M., Feng G., Ren Y., Zhang X. (2020). On Cloud Storage Optimization of Blockchain With a Clustering-Based Genetic Algorithm. IEEE Internet Things J..

[12] Li S., Xu C., Zhang Y., Du Y., Chen K. (2023). Blockchain-Based Transparent Integrity Auditing and Encrypted Deduplication for Cloud Storage. IEEE Trans. Serv. Comput..

[13] Saleem T., Ismaeel M., Janjua M.U., Ali A., Aqib A., Ahmed A., Hassan S.U. (2023). Predicting functional roles of Ethereum blockchain addresses. Peer Peer Netw. Appl..

[14] Ruan P., Kanza Y., Ooi B.C., Srivastava D. LedgerView: Access-Control Views on Hyperledger Fabric. Proceedings of the SIGMOD ‘22: International Conference on Management of Data.

[15] Wu Y., Tie G., Yu Y., Li J., Song J. (2023). EBSS: A secure blockchain-based sharing scheme for real estate financial credentials. World Wide Web.

[16] Wang L., Liu X., Shao W., Guan C., Huang Q., Xu S., Zhang S. (2024). A Blockchain-Based Privacy-Preserving Healthcare Data Sharing Scheme for Incremental Updates. Symmetry.

[17] Elisa N., Yang L., Chao F., Cao Y. (2023). A framework of blockchain-based secure and privacy-preserving E-government system. Wirel. Netw..

[18] Zhang K., Zhang Y., Li Y., Liu X., Lu L. (2024). A Blockchain-Based Anonymous Attribute-Based Searchable Encryption Scheme for Data Sharing. IEEE Internet Things J..

[19] Pei H., Yang P., Li W., Du M., Hu Z. (2024). Proxy Re-Encryption for Secure Data Sharing with Blockchain in Internet of Medical Things. Comput. Netw..

[20] Vanin F.N.D.S., Policarpo L.M., Righi R.D.R., Heck S.M., da Silva V.F., Goldim J., da Costa C.A. (2023). A Blockchain-Based End-to-End Data Protection Model for Personal Health Records Sharing: A Fully Homomorphic Encryption Approach. Sensors.

[21] Ofori A.Y., Sadat S.K., Darvishi I. Blockchain Security Encryption to Preserve Data Privacy and Integrity in Cloud Environment. Proceedings of the 10th International Conference on Future Internet of Things and Cloud, FiCloud 2023.

[22] Li L., Li Z. (2023). An Efficient Quantum Secret Sharing Scheme Based on Restricted Threshold Access Structure. Entropy.

[23] Chen F., Li Z., Jiang C., Xiang T., Yang Y. (2022). Cloud Object Storage Synchronization: Design, Analysis, and Implementation. IEEE Trans. Parallel Distrib. Syst..

[24] Hakeem S.A.A., Kim H. (2022). Centralized Threshold Key Generation Protocol Based on Shamir Secret Sharing and HMAC Authentication. Sensors.

[25] Shamsoshoara A. (2019). Overview of Blakley’s Secret Sharing Scheme. arXiv.

[26] Fotiou N., Thomas Y., Siris V.A., Xylomenos G., Polyzos G.C. (2023). Self-verifiable content using decentralized identifiers. Comput. Netw..

[27] Jang I. (2017). The Pareto principle and resource egalitarianism. Math. Soc. Sci..

[28] Kılıç B., Özturan C., Sen A. (2022). Parallel analysis of Ethereum blockchain transaction data using cluster computing. Clust. Comput..

[29] Suresh K., Anand K., Nagappan G., Pugalenthi R. (2024). A Blockchain-Based Cloud File Storage System Using Fuzzy-Based Hybrid-Flash Butterfly Optimization Approach for Storage Weight Reduction. Int. J. Fuzzy Syst..

[30] Sharma P., Namasudra S., Lorenz P. Blockchain-Based Cloud Storage System with Enhanced Optimization and Integrity Preservation. Proceedings of the ICC 2023—IEEE International Conference on Communications.

[31] Wang F., Zhou J.T. Blockchain-Based Multi-Cloud Data Storage System Disaster Recovery. Proceedings of the 2023 IEEE International Conference on Systems, Man, and Cybernetics (SMC).

[32] Liu J., Chen J., Wu J., Wu Z., Fang J., Zheng Z. (2024). Fishing for Fraudsters: Uncovering Ethereum Phishing Gangs With Blockchain Data. IEEE Trans. Inf. Forensics Secur..

[33] Hussien F.T.A., Khairi T.W.A. (2023). Performance Evaluation of AES, ECC and Logistic Chaotic Map Algorithms in Image Encryption. Int. J. Interact. Mob. Technol..

[34] Xie B., Li Q., Qian H. Weak Password Scanning System for Penetration Testing. Proceedings of the Cyberspace Safety and Security—13th International Symposium, CSS 2021.

[35] Weng C., Yang C. (2023). Reversible Data Hiding in Encrypted Image Using Multiple Data-Hiders Sharing Algorithm. Entropy.

[36] Yang D., Tsai W.-T. (2024). Linear Consensus Protocol Based on Vague Sets and Multi-Attribute Decision-Making Methods. Electronics.

[37] Jia D., Xin J., Wang Z., Guo W., Wang G. ElasticChain: Support Very Large Blockchain by Reducing Data Redundancy. Proceedings of the Web and Big Data—Second International Joint Conference, APWeb-WAIM 2018.

[38] Wang X., Ning Z., Wang W., Yang Y. (2019). Anti-conspiracy attack threshold signature model and protocol. Int. J. Wirel. Mob. Comput..

